# Comprehensive Quality Evaluation of American Ginseng for Different Parts and Abnormal Trait Based on the Major Ginsenoside Contents and Morphological Characteristics

**DOI:** 10.1155/2021/8831080

**Published:** 2021-03-24

**Authors:** Jingping Yu, Tong Xu, Haiyan Lin, Ying Lin, Jie Zhou, Yongqing Zhang

**Affiliations:** ^1^School of Integrated Traditional Chinese and Western Medicine, Binzhou Medical University, Yantai, Shandong, China; ^2^School of Biological Science and Technology, University of Jinan, Jinan, Shandong, China; ^3^School of Pharmacy, Shandong University of Traditional Chinese Medicine, Jinan, Shandong, China

## Abstract

The demand for American ginseng, a famous traditional medicine and high-grade healthy food, has increased dramatically over recent years. However, only the main root is popular among consumers, whereas other parts of American ginseng are rarely available in the market. In this study, the contents of 5 major ginsenosides (Re, Rc, Rg_1_, Rd, and Rb_1_) were determined through high-performance liquid chromatography. Our study showed that all these 5 major ginsenosides are found in different parts of American ginseng plants, and the total content in different parts varied significantly in the following order: fibrous root > flower > branch root > main root > leaf > stem. Interestingly, the total content in the fibrous root was approximately 2.24 times higher than that in the main root. Further research indicated that the ginsenoside content in American ginseng with abnormal characteristics (physical deformity caused by disease and discolouration) is similar to that in the normal plant. Interestingly, a positive correlation was observed between the main root diameter and total ginsenoside content, whereas a negative correlation was observed between the main root length and total ginsenoside content. Our comprehensive study revealed that all parts of American ginseng, including the main root with abnormal characteristics, possess medicinal or economic value. Therefore, our results provide feasible evidence to further explore the potential application of American ginseng.

## 1. Introduction

American ginseng (*Panax quinquefolius* L.) is native to North America and has been widely used as a traditional medicine and in health tonics [1]. Recent studies have reported that American ginseng exhibits immunomodulatory [2], antitumour [3], antioxidative [4], anti-inflammatory [5], and antidiabetic [6] effects, prevents ovarian ageing [7], and helps in expediting recovery from common cold [8]. Ginsenosides, as the main secondary metabolites of American ginseng, are the main bioactive components [9, 10]. Because of its multiple biological activities, American ginseng has been widely adopted as a fundamental ingredient in functional foods, pharmaceutical products, cosmetics, and nutraceuticals. Currently, artificial cultivation of American ginseng is encouraged to satisfy the ever-increasing market demands [11, 12]. However, further application of American ginseng is still restricted because of its slow growth rate, high price, and complicated quality evaluation criteria.

So far, considerable efforts have been made to resolve these problems, and one of the main strategies is to increase production and quality. For example, a small seedling size is known to be associated with a high ginsenoside content in the main root (MR) [13]. Some studies have reported that the quality of American ginseng differs considerably depending on the age and cultivation region [14, 15]. In addition, the application of biofertilisers has also shown to decrease the rate of rotten roots, thus enhancing the yield and quality [16–18]. However, most researches are focused on the root, and only the MR is popular among consumers [19]. In fact, the remaining parts such as flower, leave, stem, branch root (BR), and fibrous root (FR) are not considered a good source of ginsenoside. Thus, these parts have been rarely studied and are not available in commercial markets.

Compared with American ginseng, every part of Asian ginseng has been fully explored and medicinally utilised [20–22]. For example, the MR in the form of Chinese patent medicine is commonly used to treat cancers, fatigues, and cardiovascular diseases. The BR and FR are generally ground into powder and used as a food additive or in tonics, whereas the flower, stem, and leaf extracts are used as raw materials in cosmetic manufacturing. However, for the American ginseng, previous studies have only focused on the ginsenoside content of the MR, and therefore, the systematic profiles of the total ginsenoside contents of other parts are unknown. Additionally, the American ginseng plant is susceptible to disease, invasion by insect pests, and harsh environment during its growth, which may lead to the development of abnormal characteristics, such as physical deformity caused by disease and discolouration [23]. Normally, these abnormal roots are discarded. Therefore, it is necessary to comprehensively investigate the ginsenoside content of different parts including abnormal roots of the American ginseng, which will provide an effective solution to the complete utilisation of all the parts of this plant. Additionally, there is also lack of a simple criterion for assessing the intrinsic quality of American ginseng, which would further limit the classification according to different purposes. To date, numerous methods have been developed to determine the quality of American ginseng; however, only a few studies have focused on the correlation between the morphological characteristics of its roots and the ginsenoside content. Previous studies have reported that thin roots tend to contain a high ginsenoside content [19]. However, this finding is inconsistent with the evaluation criteria in the market, where thicker roots are costlier than thinner roots. Therefore, to conclude the debate on the relationship between quality and morphological features, further comparison of the ginsenoside content and MR morphotypes is required.

In this study, we performed a comparative analysis using the high-performance liquid chromatography (HPLC) method to investigate the possible commercial worth of 6 different parts including the abnormal root of the American ginseng plant and establish simple quality evaluation criteria for the MR. Our analysis attempts to present an overview of the ginsenoside content of American ginseng, which would serve as a reference for further research and development of potential applications of American ginseng. Moreover, the correlation between ginsenoside content and MR shapes might provide a scientific and practical standard to evaluate the quality of American ginseng based on morphological characteristics.

## 2. Materials and Methods

### 2.1. Sample Preparation

To compare differences in ginsenoside contents among different parts of American ginseng, we randomly selected 10 American ginseng (four-year-old) plants. Each plant was further divided into 6 parts: MR, BR, FR, flower, stem, and leaf. Subsequently, each part from the 10 plants was combined to form a single sample. Sixty MRs with various abnormal characteristics were collected and classified according to the abnormal characteristics into 3 grades: mild, moderate, and severe. In addition, 19 MRs (MR1–MR19) were selected to analyse the correlation between ginsenoside content and MR shapes. Sample information is listed in Table 1. The samples were washed with water, blot dried with gauzes, and placed in individual paper bags. All the samples were authenticated by the authors according to the Chinese Pharmacopoeia.

### 2.2. Preparation of Sample Solution

The samples were dried to a constant weight at 50°C and were ground, blended, and passed through sieves (50 mesh) to obtain homogeneous powder. For the 6 parts of the American ginseng and abnormal MRs, 6 powder samples from each part were prepared, and each sample weighed 1.0 g. Because of the availability of a large number of samples for the correlation analysis between ginsenoside content and MR shape, 3 copies of each sample were selected and each copy weighed 1.0 g. These samples were ultrasonically extracted for 30 min at room temperature with 50 mL of 50% methanol (Xilong Scientific Co, Ltd, Guangdong, China). The temperature of the samples increased because of ultrasonic energy. After they were cooled to room temperature, the weight loss was replenished with the same solvent. Extraction solutions were then filtered. Subsequently, 10 mL of the solutions was transferred into an evaporating dish, and the solutions were allowed to evaporate to obtain dry powder. Finally, the dried extract was dissolved in 5 mL of 50% methanol and filtered through a 0.45 *μ*m membrane before HPLC analysis [24].

### 2.3. Preparation of Reference Standards

The mixed standard solution including ginsenoside Rb_1_ (1.025 *μ*g/*μ*L), Re (0.640 *μ*g/*μ*L), Rc (0.255 *μ*g/*μ*L), Rd (0.120 *μ*g/*μ*L), and Rg_1_ (0.080 *μ*g/*μ*L) was prepared in 50% methanol. These reference standards of 5 ginsenosides (HPLC grade), Rg_1_, Re, Rb_1_, Rc, and Rd, were purchased from Shanghai Yuanye BioTech Company (Shanghai, China), and methanol was purchased from Xilong Scientific Co, Ltd (Guangdong, China).

### 2.4. Chromatographic Apparatus and Conditions

The analysis of ginsenoside content was performed using an HPLC system equipped with a binary pump (Shimadzu, LC-2030C 3D, Shimadzu Corporation, Kyoto Japan). A reverse-phase C_18_ column (4.6 mm × 250 mm, 5°*μ*m; ZORBAX ODS, Agilent, USA) was used for separation. Acetonitrile and water containing 0.05% phosphoric acid (*V*/*V*) were used as the mobile phase. The linear gradient was set as follows: 0~35°min, 19% acetonitrile; 35~55°min, 19~29% acetonitrile; 55~70°min, 29% acetonitrile; and 70~100°min, 29~40% acetonitrile (acetonitrile was purchased from Tianjin Biao Shi Qi Technology Development Co. Ltd, Tianjin, China; phosphoric acid was purchased from Tianjin Kemiou Chemical Reagent Co. Ltd, Tianjin, China). The column temperature, flow rate, and wavelength were 40°C, 1.0°mL°min^−1^, and 203 nm, respectively. This method was developed by our group based on the method provided in the Chinese Pharmacopoeia (2020), which has been verified to be reliable in analysing the ginsenoside content. Further details can be found in our previous literature [13, 16, 24].

### 2.5. Statistical Analysis

Ginsenoside content is presented as the mean ± standard deviation (*n* = 6). The analysis of variance was performed using the program SPSS 20.0, and Duncan's multiple range test was employed to analyse differences between the means. *P* value of <0.05 was considered statistically significant. For analysing correlations between the length, diameter, and ginsenoside content, the Pearson correlation coefficient test was used, and a two-tailed *P* value with a 95% confidence interval was calculated for each correlation.

## 3. Results and Discussion

### 3.1. Method Validation

**Figure 1:**
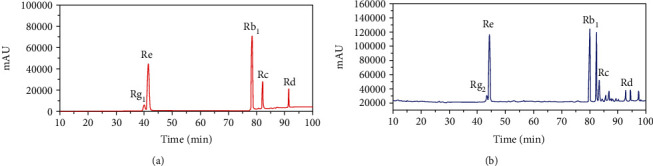
HPLC chromatograms for (a) mixture of the 5 standard ginsenosides and (b) a representative sample of American ginseng.

HPLC chromatograms of the mixed standards are shown in Figure 1(a), and all the 5 ginsenosides can be effectively distinguished on the basis of these chromatograms. The reliability of the method was validated by checking its linearity, limits of detection (LODs), limits of quantification (LOQs), precision, repeatability, stability, and recovery. All the results are listed in Table 2. To get the linear equations of the 5 ginsenosides (Rg_1_, Re, Rb_1_, Rc, and Rd), 6 levels of mixed standard solutions were obtained by diluting their stock standard solutions with 50% methanol. That is, 2, 5, 8, 10, 12, and 15 *μ*L of former standard solution and corresponding 50% methanol were taken to make 1 mL of standard solution. Then, the linearity for each component was determined by plotting the peak area (*Y*) versus the concentrations (*X*) of ginsenoside. The values of the correlation coefficient (*r* = 0.9995 ~ 0.9999) indicated a strong correlation between the measured ginsenoside contents and peak areas within test ranges. The LODs and LOQs were determined when the signal-to-noise (*S*/*N*) ratios were 3 and 10, respectively. The obtained LODs were 0.012, 0.002, 0.021, 0.027, and 0.023 *μ*g/mL for the 5 ginsenosides, respectively, while the values of LOQs were 0.022, 0.007, 0.041, 0.055, and 0.049 *μ*g/mL for the 5 ginsenosides, respectively. Precision was evaluated by analysing the peak area variations of the mixed standard solution with 6 replicated analyses. Repeatability testing was performed by analysing 6 individual samples. Stability was examined on a sample solution at time intervals of 0, 2, 4, 6, 10, and 12 h. All the RSDs of precision, repeatability, and stability were found to be less than 3.000%. Recovery assessment was evaluated using spiked samples by adding 200 *μ*L of the standard mixture into 200 *μ*L of the sample solution. This process will be repeated 6 times with 10 *μ*L for each injection. The average recovery rate was 98.522%, 100.847%, 105.009%, 104.507%, and 105.424% for Rg_1_, Re, Rb_1_, Rc, and Rd, respectively, with the RSDs of 3.096%, 1.387%, 0.716%, 1.174%, and 1.051% for each ginsenoside, respectively. These results indicate that this method is accurate enough to detect the content of ginsenoside.

### 3.2. Comparison of the 5 Major Ginsenosides in Different Parts of the American Ginseng

**Figure 2:**
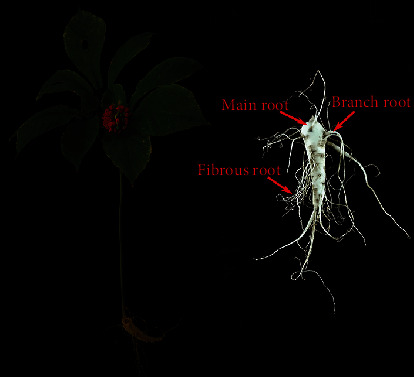
Typical morphologies of field-cultivated American ginseng (left); morphological characteristics of the main root, branch root, and fibrous root of the American ginseng plant (right).

By using the aforementioned HPLC method, we determined the ginsenoside content in MR, BR, FR, flower, stem, and leaf (Figure 2). Because the major ginsenosides (Rg_1_, Re, Rb_1_, Rc, and Rd) typically account for more than 70% of the total ginsenoside content in the American ginseng plant, these 5 major ginsenosides were considered quality indicators in this study [25, 26].  Table 3 presents the ginsenoside content of different parts.

**Figure 3:**
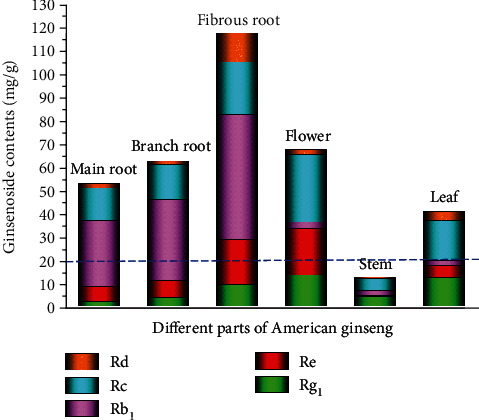
Ginsenoside contents in 6 parts of the American ginseng. The blue dotted line represents the content specified in the Chinese Pharmacopoeia (2020).

Surprisingly, all the 5 ginsenosides were detected in the 6 studied parts of the American ginseng plant. Nevertheless, a significant difference between these parts was observed in the ginsenoside content. As illustrated in Figure 3, the total ginsenoside content of different parts ranked as follows: FR > flower > BR > MR > leaf > stem. The FR had the highest level of 117.62 mg/g, which was about 1.74, 1.87, 2.24, 2.86, and 8.98 times higher than those in the flower, BR, MR, leaf, and stem, respectively. Moreover, the total ginsenoside contents in the FR and BR were more than 2.24 and 1.20 times higher than those in the MR, which is inconsistent with traditional experience. This result might be attributed to the fact that the FR is much thinner than the MR, and thus, the more periderm contributes to a higher ratio in the FR. In general, consumers believe that the MR is more effective than the BR and FR, which leads to a significant price difference among these 3 parts. However, our analysis suggests that the American ginseng plant with a thin root is of superior quality for medicinal use.

Similar to the total ginsenoside content, a large variation in individual ginsenoside amounts was observed among the 6 parts of the American ginseng. For example, the contents of Rb_1_ in the MR, BR, and FR were obviously higher than those in the flower, stem, and leaf; thus, this could be used as a major indication to identify the roots of American ginseng plants. The flower contained the highest ginsenoside contents of Rc, Re, and Rg_1_, whereas the FR contained the highest level of Rb_**1**_. Because the individual ginsenoside exhibits specific effects on different diseases, these 6 parts of the American ginseng can be used purposefully [27].

We noticed a similar trend in the Panax ginseng, wherein the FR had a higher ginsenoside content than the MR [28, 29]. However, compared with the American ginseng, obvious differences were found in the flower, leaf, and stem of the Panax ginseng. The decreasing order of the total ginsenoside content in these 3 parts of the Panax ginseng was as follows: leaf > flower > stem. Additionally, not all the 5 individual ginsenosides could be detected in the stem and leaf. The Rb_1_ contents in the stem and leaves were considerably low, and Rc was almost not detected in the stem of the Panax ginseng. Nevertheless, the contents of the ginsenoside Rc in leaf and stem were compared to the ones in the root for the American ginseng. These results indicated that the American and Panax ginsengs should be used differently according to the requirement.

The sum of the individual ginsenoside (Rg_1_, Re, and Rb_1_) content in the American ginseng, which is the index stipulated in the Chinese Pharmacopoeia (2020), has also been reported. As presented in Figure 3 and Table S1, the total of Rg_1_, Re, and Rb_1_ content in different parts, except for the stem, was higher than the Pharmacopoeia standard (>20 mg/g). The result indicated that not only the MR but also the BR, FR, flower, and leaf can be used as traditional Chinese medicine. Although the sum of Rg_1_, Re, and Rb_1_ in the stem was lower than the pharmaceutical standards, it could also be used as a raw material in cosmetic manufacturing. In the past, these parts were often discarded, which resulted in great losses. We can fully utilise the flower, stem, and leaf as raw materials for extracting active ingredients and for developing means to maximise the utilisation of traditional Chinese medicine resources. Moreover, although the FR is not as popular as the MR in the medicinal material market, it had the highest ginsenoside content among the 6 parts of American ginseng that were investigated, which means that more attention should be paid to its medicinal value in the future. Therefore, all 6 different parts of the American ginseng could be effectively used in various products according to different needs, and our study could provide an important visual standardisation for quality evaluation and classification of American ginseng plants.

### 3.3. Comparison of the 5 Major Ginsenosides in the American Ginseng with Various Abnormal Characteristics

To date, only a few studies have focused on determining the correlation between abnormal characteristics and the quality of American ginseng. Although the parts of *Salvia miltiorrhiza* with abnormal characteristics, such as physical deformity caused by disease and discolouration, still contain comparable active components compared with the healthy parts [30], the same cannot be concluded for the American ginseng. Therefore, the 5 major ginsenosides were also detected in the abnormal MRs. The results are listed in Table 3.

**Figure 4:**
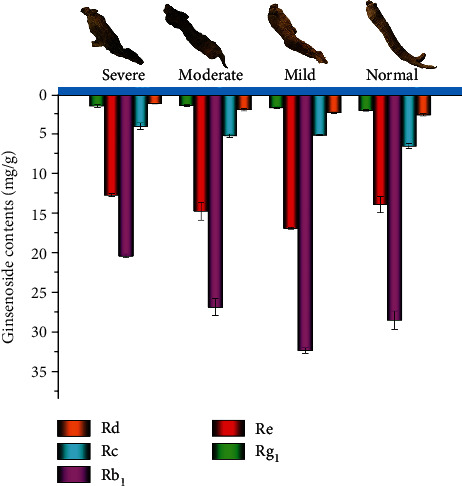
Ginsenoside contents of the American ginseng plant with different degrees of abnormal characteristics.

Surprisingly, no significant difference was observed in the ginsenoside content among the 4 grades of the American ginseng, as shown in Figure 4. The total ginsenoside content of these MRs was ranked as mild > normal > moderate > severe, where the values were 58.00, 52.45, 49.78, and 39.30 mg/g, respectively. A similar trend was also observed for the individual ginsenoside content. Notably, the abnormal American ginseng of the mild grade displayed the highest ginsenoside content. This phenomenon may be caused by the change in metabolism due to diseases, insect pests, and adverse environmental conditions, and it has also been reported in other plants such as *Salvia miltiorrhiza* [30], *Panax notoginseng* [31], and *Glycyrrhiza uralensis* [32].

The sum of individual ginsenoside (Rg_1_, Re, and Rb_1_) contents was 34.35, 42.83, and 50.79 mg/g for the severe, moderate, and mild ones, respectively. Obviously, the sum of individual ginsenoside (Rg_1_, Re, and Rb_1_) contents was higher than the Chinese Pharmacopoeia standard (20 mg/g) (2020). Therefore, the American ginseng with abnormal characteristics should be effectively used rather than being discarded.

### 3.4. Correlation between Morphological Characteristics and Ginsenoside Contents

Presently, the MR is the most common part of the American ginseng plant that is sold in the market. Thus, the exploration of the relationship between its quality and morphological features is essential. In this study, we analysed 19 MR samples of the American ginseng, as shown in Figure S2. Among the morphological characteristics, the diameter and length of the MR were determined as a simple and objective measurement index. The quantitative results between the 5 major ginsenoside contents and their morphological characteristics are listed in Tables 1 and 3.

**Figure 5:**
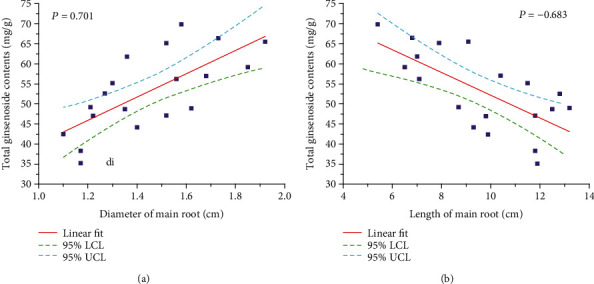
(a) Correlation between diameter and total ginsenoside contents of the American ginseng. (b) Correlation between length and total ginsenoside content of the American ginseng.

The diameter of all the MRs ranged from 1.10 cm to 1.92 cm, whereas the length ranged from 5.4 cm to 13.2 cm. The 5 ginsenoside contents varied significantly in the MRs with different diameters and lengths. The total ginsenoside contents ranged from 35.15 to 69.88 mg/g, and the average value was 53.16 mg/g. This result was consistent with that reported in Section 3.2, which confirms the accuracy of the method adopted in the present study. The correlation between the total ginsenoside content and morphological characteristics is shown in Figure 5. Interestingly, an opposite trend was observed for the diameter and length. The Pearson correlation coefficient was 0.701 for the diameter, whereas the value was −0.683 for the length. These results indicate that the MRs with thicker and shorter morphology contain higher levels of ginsenosides, which is consistent with the evaluation criteria in the market where thicker MRs have higher prices. It indicates that the diameter and length of the MR are key morphological characteristics and can be used by consumers and retailers to roughly estimate the quality of the American ginseng. Consequently, dividing the MR of the American ginseng plant into specific grades and optimising the utilisation of each grade will greatly enhance the production value and improve economic benefits rather than mixing them together.

## 4. Conclusion

In summary, this study provides an overview of the inherent qualities of the American ginseng using the HPLC method. Our findings indicate that the ginsenoside content of the American ginseng has a strong correlation with the parts, degree of abnormal characteristics, and morphological characteristics. The MR, BR, FR, flower, stem, and leaf are all rich in the 5 major ginsenosides, namely, Rg_1_, Re, Rb_1_, Rc, and Rd. Moreover, the ginsenoside contents in all the parts, except for the stem, meet the pharmacopoeia standard. The ginsenoside contents in the abnormal MRs were compared to those in the normal MRs, and no obvious difference was found. In addition, our results indicate that a thicker MR contains greater amounts of ginsenosides, but a longer MR contains lower amounts of ginsenosides. Our study provides scientific evidence for further development and for the effective and economic use of every part of American ginseng plants.

## Figures and Tables

**Table 1:** Sample information.

Type	Sample	Production	Origin	Collection date	Age (years)	Morphological features
Different parts	MR	Cultivated, fresh	Wendeng, Shandong (40°58′21^″^N, 125°56′28^″^E)	November 6, 2018	4	Texture hard and heavy. 5.0-13.5 cm (length), 1.10-2.10 cm (diameter), 5.0-16.0 g (weight).
	BR	Cultivated, fresh	Wendeng, Shandong (40°58′21^″^N, 125°56′28^″^E)	November 6, 2018	4	Texture hard and heavy. 2.7-4.7 cm (length), 0.30-0.53 cm (diameter), 0.23-0.61 g (weight).
	FR	Cultivated, fresh	Wendeng, Shandong (40°58′21^″^N, 125°56′28^″^E)	November 6, 2018	4	5.7-13.2 cm (length), 0.07-0.15 cm (diameter).
	Flower	Cultivated, fresh	Wendeng, Shandong (40°58′21^″^N, 125°56′28^″^E)	November 6, 2018	4	Inflorescence a solitary, terminal umbel 6-20-flowered; peduncle not exceeding petioles.
	Stem	Cultivated, fresh	Wendeng, Shandong (40°58′21^″^N, 125°56′28^″^E)	November 6, 2018	4	Herbs, perennial, 20.0-50.0 cm tall.
	Leaf	Cultivated, fresh	Wendeng, Shandong (40°58′21^″^N, 125°56′28^″^E)	November 6, 2018	4	Leaves palmately compound; leaflets oblong-obovate membranous, margin coarsely serrate or dentate, apex abruptly or boldly acuminate.
Main roots with abnormal characteristics	Mild	Cultivated, dried	Wendeng, Shandong (40°58′21^″^N, 125°56′28^″^E)	November 6, 2018	4	Root epidermis blackened but subcutaneous tissue normal.
	Moderate	Cultivated, dried	Wendeng, Shandong (40°58′21^″^N, 125°56′28^″^E)	November 6, 2018	4	Root center decays with black cavities.
	Severe	Cultivated, dried	Wendeng, Shandong (40°58′21^″^N, 125°56′28^″^E)	November 6, 2018	4	Root rot and blackening.
Main roots with different morphological characteristics	MR1	Cultivated, dried	Wendeng, Shandong (40°58′21^″^N, 125°56′28^″^E)	December 14, 2017	4	Texture hard and heavy. 10.4 cm (length), 1.68 cm (diameter), 13.97 g (weight).
	MR2	Cultivated, dried	Wendeng, Shandong (40°58′21^″^N, 125°56′28^″^E)	December 14, 2017	4	Texture hard and heavy. 9.1 cm (length), 1.92 cm (diameter), 12.90 g (weight).
	MR3	Cultivated, dried	Wendeng, Shandong (40°58′21^″^N, 125°56′28^″^E)	December 14, 2017	4	Texture hard and heavy. 11.8 cm (length), 1.52 cm (diameter), 15.86 g (weight).
	MR4	Cultivated, dried	Wendeng, Shandong (40°58′21^″^N, 125°56′28^″^E)	December 14, 2017	4	Texture hard and heavy. 9.3 cm (length), 1.40 cm (diameter), 13.96 g (weight).
	MR5	Cultivated, dried	Wendeng, Shandong (40°58′21^″^N, 125°56′28^″^E)	December 14, 2017	4	Texture hard and heavy. 7.1 cm (length), 1.56 cm (diameter), 10.37 g (weight).
	MR6	Cultivated, dried	Wendeng, Shandong (40°58′21^″^N, 125°56′28^″^E)	December 14, 2017	4	Texture hard and heavy. 13.2 cm (length), 1.62 cm (diameter), 14.26 g (weight)
	MR7	Cultivated, dried	Wendeng, Shandong (40°58′21^″^N, 125°56′28^″^E)	December 14, 2017	4	Texture hard and heavy. 7.9 cm (length), 1.52 cm (diameter), 10.21 g (weight).
	MR8	Cultivated, dried	Wendeng, Shandong (40°58′21^″^N, 125°56′28^″^E)	December 14, 2017	4	Texture hard and heavy. 6.5 cm (length), 1.85 cm (diameter), 9.72 g (weight)
	MR9	Cultivated, dried	Wendeng, Shandong (40°58′21^″^N, 125°56′28^″^E)	December 14, 2017	4	Texture hard and heavy. 5.4 cm (length), 1.58 cm (diameter), 8.25 g (weight).
	MR10	Cultivated, dried	Wendeng, Shandong (40°58′21^″^N, 125°56′28^″^E)	December 14, 2017	4	Texture hard and heavy. 6.8 cm (length), 1.73 cm (diameter), 12.04 g (weight).
	MR11	Cultivated, dried	Wendeng, Shandong (40°58′21^″^N, 125°56′28^″^E)	December 14, 2017	4	Texture hard and heavy. 7.0 cm (length), 1.36 cm (diameter), 7.75 g (weight).
	MR12	Cultivated, dried	Wendeng, Shandong (40°58′21^″^N, 125°56′28^″^E)	December 14, 2017	4	Texture hard and heavy. 11.5 cm (length), 1.30 cm (diameter), 5.89 g (weight).
	MR13	Cultivated, dried	Wendeng, Shandong (40°58′21^″^N, 125°56′28^″^E)	December 14, 2017	4	Texture hard and heavy. 8.7 cm (length), 1.21 cm (diameter), 5.66 g (weight).
	MR14	Cultivated, dried	Wendeng, Shandong (40°58′21^″^N, 125°56′28^″^E)	December 14, 2017	4	Texture hard and heavy. 11.8 cm (length), 1.17 cm (diameter), 6.04 g (weight).
	MR15	Cultivated, dried	Wendeng, Shandong (40°58′21^″^N, 125°56′28^″^E)	December 14, 2017	4	Texture hard and heavy. 9.9 cm (length), 1.10 cm (diameter), 5.92 g (weight).
	MR16	Cultivated, dried	Wendeng, Shandong (40°58′21^″^N, 125°56′28^″^E)	December 14, 2017	4	Texture hard and heavy. 9.8 cm (length), 1.22 cm (diameter), 6.09 g (weight).
	MR17	Cultivated, dried	Wendeng, Shandong (40°58′21^″^N, 125°56′28^″^E)	December 14, 2017	4	Texture hard and heavy. 11.9 cm (length), 1.17 cm (diameter), 7.43 g (weight).
	MR18	Cultivated, dried	Wendeng, Shandong (40°58′21^″^N, 125°56′28^″^E)	December 14, 2017	4	Texture hard and heavy. 12.8 cm (length), 1.27 cm (diameter), 7.93 g (weight).
	MR19	Cultivated, dried	Wendeng, Shandong (40°58′21^″^N, 125°56′28^″^E)	December 14, 2017	4	Texture hard and heavy. 12.5 cm (length), 1.35 cm (diameter), 7.47 g (weight).

**Table 2:** The data for method validation.

Ginsenosides	Calibration curve	Correlation coefficient (*r*)	Linearity range (*μ*g/mL)	Repeatability (*n* = 6, RSD, %)	Stability (12 h, RSD, %)	LOD (*μ*g/mL)	LOQ (*μ*g/mL)	Precision (*n* = 6, RSD, %)	Recovery (*n* = 6, RSD, %)	
									Mean	RSD
Rg_1_	*y* = 349649.00*x* − 7902.50	0.9995	0.162~1.214	2.310	0.840	0.012	0.022	1.311	98.522	3.096
Re	*y* = 269839.67*x* − 13,991.90	0.9999	1.281~9.627	1.801	0.461	0.002	0.007	1.720	100.847	1.387
Rb_1_	*y* = 167043.17*x* − 3939.96	0.9999	2.052~15.379	1.722	1.172	0.021	0.041	2.101	105.009	0.716
Rc	*y* = 200211.36*x* − 2882.38	0.9999	0.513~3.827	0.913	0.640	0.027	0.055	1.923	104.507	1.174
Rd	*y* = 244430.95*x* − 1949.91	0.9999	0.240~1.824	2.920	1.921	0.023	0.049	2.420	105.424	1.051

**Table 3:** Ginsenoside content in different American ginseng samples (mg/g).

Type	Sample	Rg_**1**_	Re	Rb_**1**_	Rc	Rd	Rg_1_+Re+Rb_1_	Rg_1_+Re+Rb_1_+Rc+Rd
Main roots with abnormal characteristics	Normal	1.89 ± 0.21	13.56 ± 0.92	28.10 ± 2.56	6.43 ± 0.57	2.47 ± 0.63	43.55	52.45
	Severe	1.34 ± 0.17	12.64 ± 0.19	20.37 ± 0.25	3.92 ± 0.42	1.03 ± 0.01	34.35	39.30
	Moderate	1.29 ± 0.08	14.67 ± 1.13	26.87 ± 1.97	5.13 ± 0.23	1.82 ± 0.14	42.83	49.78
	Mild	1.57 ± 0.02	16.85 ± 0.02	32.37 ± 0.34	5.02 ± 0.11	2.19 ± 0.10	50.79	58.00
Main roots with different morphological characteristics	MR1	0.61 ± 0.02	14.27 ± 1.10	28.65 ± 2.01	8.81 ± 0.60	4.63 ± 0.10	43.54	56.98
	MR2	2.35 ± 0.11	20.64 ± 1.09	30.53 ± 0.95	8.55 ± 0.53	3.50 ± 0.80	53.53	65.57
	MR3	0.20 ± 0.01	8.34 ± 1.02	32.85 ± 1.60	4.01 ± 0.41	1.68 ± 0.04	41.38	47.07
	MR4	0.64 ± 0.03	12.32 ± 1.00	23.32 ± 1.93	6.32 ± 0.67	1.59 ± 0.08	36.29	44.20
	MR5	0.25 ± 0.03	9.73 ± 1.03	39.18 ± 1.70	3.77 ± 0.04	3.35 ± 0.22	49.16	56.27
	MR6	1.46 ± 0.05	14.12 ± 1.18	25.11 ± 1.66	6.57 ± 0.50	1.75 ± 0.08	40.69	49.01
	MR7	0.42 ± 0.07	16.25 ± 1.30	37.71 ± 1.58	7.84 ± 0.87	2.97 ± 0.21	54.38	65.19
	MR8	0.57 ± 0.01	20.23 ± 1.25	33.94 ± 0.80	3.41 ± 0.59	1.04 ± 0.04	54.74	59.20
	MR9	0.89 ± 0.02	18.85 ± 1.30	39.48 ± 1.60	8.34 ± 0.26	2.33 ± 0.13	59.22	69.88
	MR10	0.47 ± 0.01	12.44 ± 1.51	42.53 ± 1.97	6.06 ± 0.65	4.99 ± 0.17	55.44	66.49
	MR11	0.49 ± 0.02	14.57 ± 1.63	32.20 ± 1.95	10.40 ± 0.58	4.05 ± 0.08	47.26	61.71
	MR12	0.52 ± 0.04	16.09 ± 1.50	25.38 ± 1.60	12.12 ± 0.43	1.10 ± 0.05	41.99	55.21
	MR13	0.82 ± 0.03	14.50 ± 1.22	24.27 ± 1.56	7.61 ± 0.89	1.99 ± 0.17	39.58	49.19
	MR14	0.80 ± 0.02	14.42 ± 1.30	14.41 ± 1.57	6.61 ± 0.59	2.05 ± 0.19	29.62	38.28
	MR15	0.29 ± 0.01	9.65 ± 1.02	25.50 ± 1.69	3.66 ± 0.84	3.30 ± 0.08	35.44	42.40
	MR16	0.50 ± 0.03	13.88 ± 1.16	24.07 ± 1.64	6.85 ± 0.80	1.68 ± 0.61	38.45	46.98
	MR17	0.76 ± 0.05	7.97 ± 1.04	19.25 ± 0.98	5.15 ± 0.65	2.03 ± 0.52	27.98	35.15
	MR18	0.37 ± 0.03	13.24 ± 1.50	30.14 ± 0.87	7.70 ± 0.41	1.17 ± 0.79	47.76	52.62
	MR19	0.60 ± 0.01	19.00 ± 1.20	23.41 ± 1.40	3.41 ± 0.58	2.25 ± 0.98	43.01	48.67

## Data Availability

All data generated or analysed during this study are included in this published article. The datasets used or analysed during the current study are available from the corresponding authors on reasonable request.

## Supplementary Materials

Supplementary Materials Figure S1. Typical morphologies of the main roots with different abnormal characteristics. Figure S2. Morphological characteristics of the 19 samples for main roots, Figure S3. The representative HPLC chromatograms for all the different plant tissues of American ginseng, Table S1. Ginsenoside contents in different parts of American ginseng (mg/g).

## References

[1] Nadeau I., Simard R. R., Olivier A. (2003). The impact of lime and organic fertilization on the growth of wild-simulated American ginseng. *Canadian Journal of Plant Science*.

[2] Zhu W., Han B., Sun Y., Wang Z., Yang X. (2012). Immunoregulatory effects of a glucogalactan from the root of _Panax quinquefolium_ L.. *Carbohydrate Polymers*.

[3] Duda R. B., Zhong Y., Navas V., Li M. Z. C., Toy B. R., Alavarez J. G. (1999). American ginseng and breast cancer therapeutic agents synergistically inhibit MCF-7 breast cancer cell growth. *Journal of Surgical Oncology*.

[4] Kitts D. D., Wijewickreme A. N., Hu C. (2000). Antioxidant properties of a North American ginseng extract. *Molecular and Cellular Biochemistry*.

[5] Qi L. W., Wang C. Z., Yuan C. S. (2011). Ginsenosides from American ginseng: chemical and pharmacological diversity. *Phytochemistry*.

[6] Vuksan V., Sievenpiper J. L., Wong J. (2001). American ginseng (Panax quinquefolius L.) attenuates postprandial glycemia in a time-dependent but not dose-dependent manner in healthy individuals. *American Journal of Clinical Nutrition*.

[7] Liao D., Jia C., Sun P., Qi J., Li X. (2019). Quality evaluation of _Panax quinquefolium_ from different cultivation regions based on their ginsenoside content and radioprotective effects on irradiated mice. *Scientific Reports*.

[8] Assinewe V. A., Baum B. R., Gagnon D., Arnason J. T. (2003). Phytochemistry of wild populations of Panax quinquefolius L. (North American ginseng). *Journal of Agricultural and Food Chemistry*.

[9] Lee D. G., Lee J. S., Kim K.-T., Kim H. Y., Lee S. (2019). Analysis of major ginsenosides in various ginseng samples. *Journal of Applied Biological Chemistry*.

[10] Kochan E., Chmiel A. (2013). Callus of American ginseng (Panax quinquefolius) as a source of ginsenosides-medicinal secondary metabolites. *Biological Letters*.

[11] Lin H., Zhu H., Tan J. (2019). Comprehensive investigation on metabolites of wild-simulated American ginseng root based on ultra-high-performance liquid chromatography-quadrupole time-of-flight mass spectrometry. *Journal of Agricultural and Food Chemistry*.

[12] Dai Y. L., Qiao M. D., Yu P., Zheng F., Yue H., Liu S. Y. (2020). Comparing eight types of ginsenosides in ginseng of different plant ages and regions using RRLC-Q-TOF MS/MS. *Journal of Ginseng Research*.

[13] Zhang H., Xu S., Pang S., Piao X., Wang Y. (2018). Effect of seed size on seedling performance, yield and ginsenoside content ofPanax ginseng. *Seed Science and Technology*.

[14] Xia Y.-G., Song Y., Liang J., Guo X.-D., Yang B.-Y., Kuang H.-X. (2018). Quality analysis of American ginseng cultivated in Heilongjiang using UPLC-ESI(-)-MRM-MS with chemometric methods. *Molecules*.

[15] Qu C., Bai Y., Jin X. (2009). Study on ginsenosides in different parts and ages of _Panax quinquefolius_ L.. *Food Chemistry*.

[16] Dong L., Li Y., Xu J. (2019). Biofertilizers regulate the soil microbial community and enhance Panax ginseng yields. *Chinese Medicine*.

[17] Liu N., Shao C., Sun H. (2020). Arbuscular mycorrhizal fungi biofertilizer improves American ginseng ( _Panax quinquefolius_ L.) growth under the continuous cropping regime. *Geoderma*.

[18] Jiao X.-L., Zhang X.-S., Lu X.-H., Qin R., Bi Y.-M., Gao W.-W. (2019). Effects of maize rotation on the physicochemical properties and microbial communities of American ginseng cultivated soil. *Scientific Reports*.

[19] Chen Y., Zhao Z., Chen H. (2017). Determination of ginsenosides in Asian and American ginsengs by liquid chromatography-quadrupole/time-of-flight MS: assessing variations based on morphological characteristics. *Journal of Ginseng Research*.

[20] Chen L.-x., Qi Y.-l., Qi Z. (2019). A comparative study on the effects of different parts of Panax ginseng on the immune activity of cyclophosphamide-induced immunosuppressed mice. *Molecules*.

[21] Zhang F., Tang S., Zhao L. (2021). Stem-leaves of Panax as a rich and sustainable source of less-polar ginsenosides: comparison of ginsenosides from _Panax ginseng,_ American ginseng and _Panax notoginseng_ prepared by heating and acid treatment. *Journal of Ginseng Research*.

[22] Li K. K., Li S. S., Xu F., Gong X. J. (2020). Six new dammarane-type triterpene saponins from _Panax ginseng_ flower buds and their cytotoxicity. *Journal of Ginseng Research*.

[23] DesRochers N., Walsh J. P., Renaud J. B., Seifert K. A., Yeung K. K.-C., Sumarah M. W. (2020). Metabolomic profiling of fungal pathogens responsible for root rot in American ginseng. *Metabolites*.

[24] Yu J. P., Liu S. J., Li L. M. (2019). Comparison of ginsenosides from Panaxquinquefolium L. by two different extraction methods. *Journal of Binzhou Medical University*.

[25] Jia L., Zhao Y. (2009). Current evaluation of the millennium phytomedicine- ginseng (I):etymology, pharmacognosy, phytochemistry, market and regulations. *Current Medicinal Chemistry*.

[26] Schlag E. M., McIntosh M. S. (2006). Ginsenoside content and variation among and within American ginseng ( _Panax quinquefolius_ L.) populations. *Phytochemistry*.

[27] Go G. Y., Jo A., Seo D. W. (2020). Ginsenoside Rb_1_ and Rb_2_ upregulate Akt/mTOR signaling-mediated muscular hypertrophy and myoblast differentiation. *Journal of Ginseng Research*.

[28] Lee J., Choi B.-R., Kim Y.-C. (2017). Comprehensive profiling and quantification of ginsenosides in the root, stem, leaf, and berry of Panax ginseng by UPLC-QTOF/MS. *Molecules*.

[29] SHI W., WANG Y., LI J., ZHANG H., DING L. (2007). Investigation of ginsenosides in different parts and ages of _Panax ginseng_. *Food Chemistry*.

[30] Li C., Wei G. F., Li J., Zheng L. M., Zhang Y. Q. (2014). Research on the utilization value of Salvia miltiorrhiza with abnormal characters. *Shandong Science*.

[31] Shang J. H., Sun W. J., Zhu H. T., Wang D., Yang C. R., Zhang Y. J. (2020). New hydroperoxylated and 20,24-epoxylated dammarane triterpenes from the rot roots of _Panax notoginseng_. *Journal of Ginseng Research*.

[32] Li Q., Gao H. Y., Guo J. F. (2019). Research progress on main chemical composition and pharmacodynami activity of Glycyrrhiza uralensi relationship with environment. *Heilongjiang Agricultural Sciences*.

