# Supplementary material for: Comprehensive Quality Evaluation of American Ginseng for Different Parts and Abnormal Trait Based on the Major Ginsenoside Contents and Morphological Characteristics
Source: Biomed Res Int. 2021 Mar 24;2021:8831080. doi: 10.1155/2021/8831080 (PMC8016571; doi:10.1155/2021/8831080)
Supplement: Supplementary Materials — Figure S1. Typical morphologies of the main roots with different abnormal characteristics. Figure S2. Morphological characteristics of the 19 samples for main roots, Figure S3. The representative HPLC chromatograms for all the different plant tissues of American ginseng, Table S1. Ginsenoside contents in different parts of American ginseng (mg/g). [file 8831080.f1.docx]

*Supplementary Information for*

**Comprehensive quality evaluation of American ginseng for different parts based on the major ginsenoside contents and morphological characteristics**

Jingping Yu^1,^*, Tong Xu^1^, Haiyan Lin^1^, Ying Lin^1^, Jie Zhou^2^, Yongqing Zhang^3,^*

^1^ *School of Integrated Traditional Chinese and Western Medicine, Binzhou Medical University, Yantai, Shandong, China*

^2^ *School of Biological Science and Technology, University of Jinan, Jinan, Shandong, China*

^3^ *School of pharmacy, Shandong University of Traditional Chinese medicine, Jinan, Shandong, China*

**To whom correspondence should be addressed.**

^*^Email: yujingping69@163.com; [zyq622003@126.com](mailto:zyq622003@126.com).


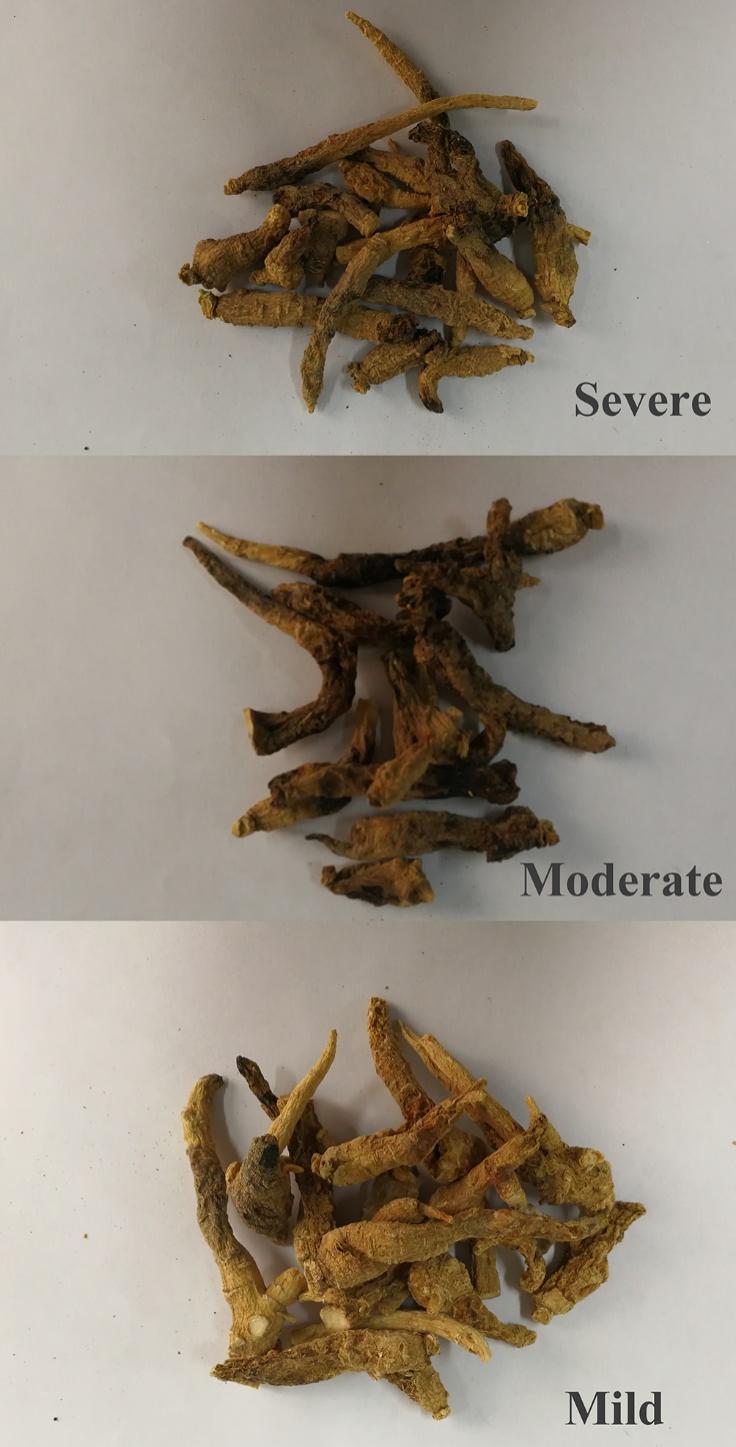


**Figure S1.** Typical morphologies of the main roots with different abnormal characteristics.


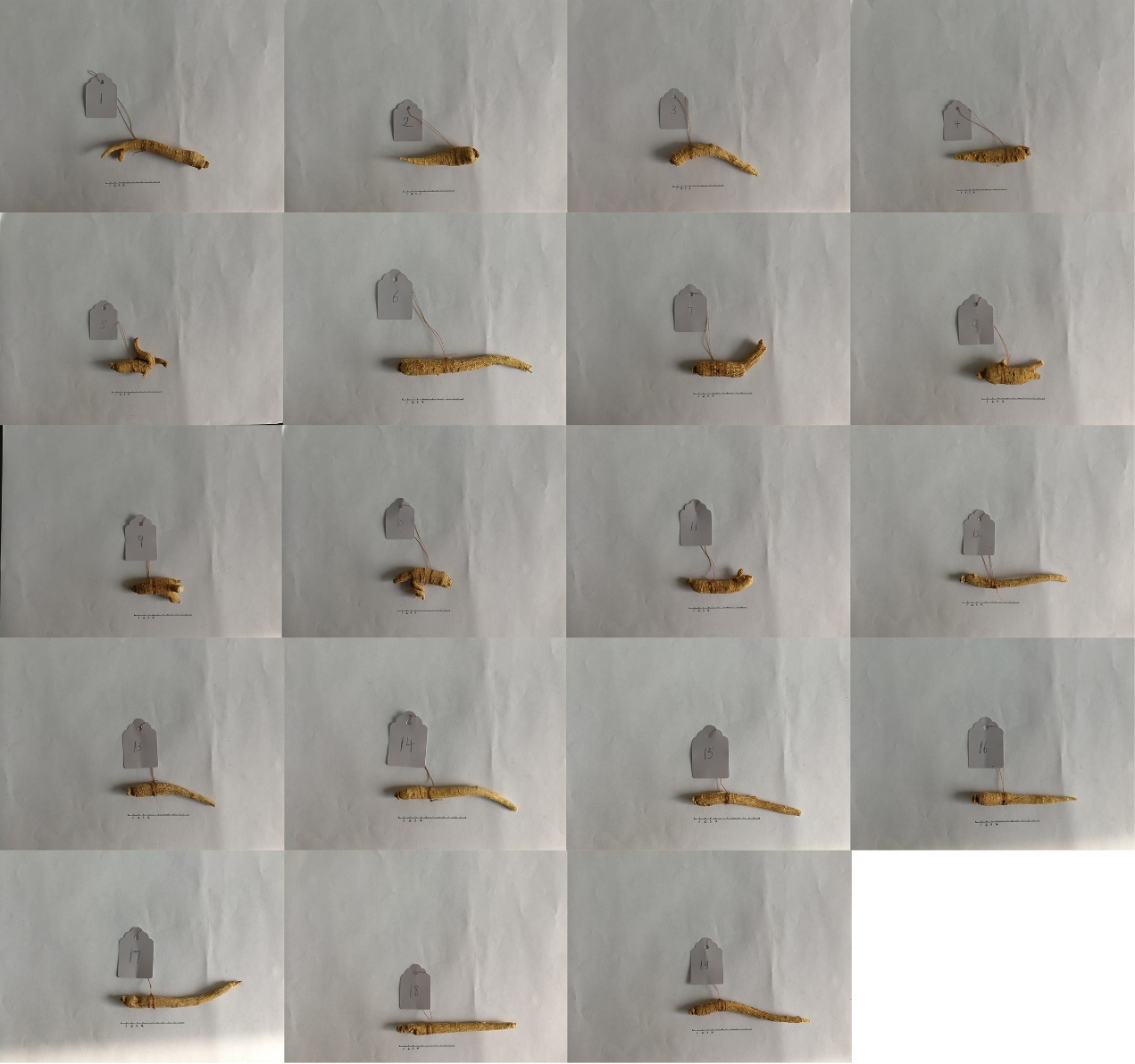


**Figure S2.** Morphological characteristics of the 19 samples for main roots.


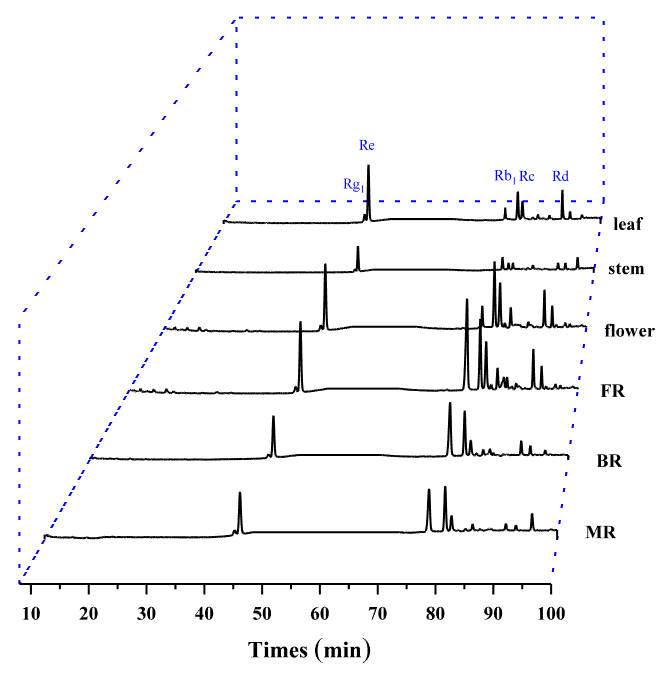


**Figure S3.** The representative HPLC chromatograms for all the different plant tissues of American ginseng.

**Table S1.** Ginsenoside contents in different parts of American ginseng (mg/g).

| Type | Sample | Rg1 | Re | Rb1 | Rc | Rd | Rg_1_+Re+Rb_1_ | Rg_1_+Re+Rb_1_  +Rc+Rd |
| --- | --- | --- | --- | --- | --- | --- | --- | --- |
| Different parts | MR | 1.89±0.21 | 13.56±0.92 | 28.10±2.56 | 6.43±0.57 | 2.47±0.63 | 43.55 | 52.45 |
|  | BR | 1.23±0.28 | 15.06±1.30 | 34.86±3.95 | 7.47±1.62 | 4.19±0.93 | 51.15 | 62.81 |
|  | FR | 12.44±1.64 | 22.24±0.05 | 53.90±0.82 | 19.02±0.26 | 10.01±0.15 | 88.59 | 117.62 |
|  | flower | 2.00±0.07 | 29.00±0.32 | 3.00±0.12 | 19.80±0.52 | 13.90±0.09 | 34.00 | 67.70 |
|  | stem | 0.80±0.01 | 5.10±0.4 | 2.10±0.02 | 0.50±0.01 | 4.60±0.52 | 8.00 | 13.10 |
|  | leaf | 3.80±0.12 | 17.20±0.65 | 2.00±0.05 | 5.10±0.05 | 13.00±0.23 | 23.00 | 41.10 |
